# Correction: Mask family proteins ANKHD1 and ANKRD17 regulate YAP nuclear import and stability

**DOI:** 10.7554/eLife.113290

**Published:** 2026-10-16

**Authors:** Clara Sidor, Nerea Borreguero-Munoz, Georgina C Fletcher, Ahmed Elbediwy, Oriane Guillermin, Barry J Thompson

**Keywords:** *D. melanogaster*

Sidor C, Borreguero-Munoz N, Fletcher GC, Elbediwy A, Guillermin O, Thompson BJ. 2019. Mask family proteins ANKHD1 and ANKRD17 regulate YAP nuclear import and stability. *eLife* **8**:e48601. doi: 10.7554/eLife.48601.

Published 29 October 2019

We were notified by eLife and PubPeer that this article contained images that had previously been published by our group in Elbediwy et al. (2016; DOI: 10.1242/dev.133728).

We identified four duplicated images, all in Figure 8A. Figure 8A was intended to show that the subcellular localisation of YAP closely follows that of Mask1 and Mask2 in response to different stimuli.

During the experiments used for the 2016 publication, cells were co-stained for YAP, Mask1, and Mask2; however, only the YAP channel was ultimately included in that publication. While preparing Figure 8A for the present article, which examined YAP localisation together with Mask1 and Mask2 localisation under various conditions, four images from these original YAP/Mask1/Mask2 triple-staining experiments were inadvertently selected. The YAP, Mask1, and Mask2 channels from these images were included in Figure 8A. Consequently, the four YAP staining panels in Figure 8A duplicated panels published in the 2016 article.

The duplicated images have now been replaced with images from a separate experiment conducted during the same period of data collection. These replacement images were not included in the 2016 publication. The duplication resulted from an inadvertent error. Because the duplicated images were representative images and were not used for quantitative analysis, their replacement does not affect the results, interpretation, or conclusions of the article.

The corrected Figure 8 (panel A updated) is shown here:

**Figure 1:**
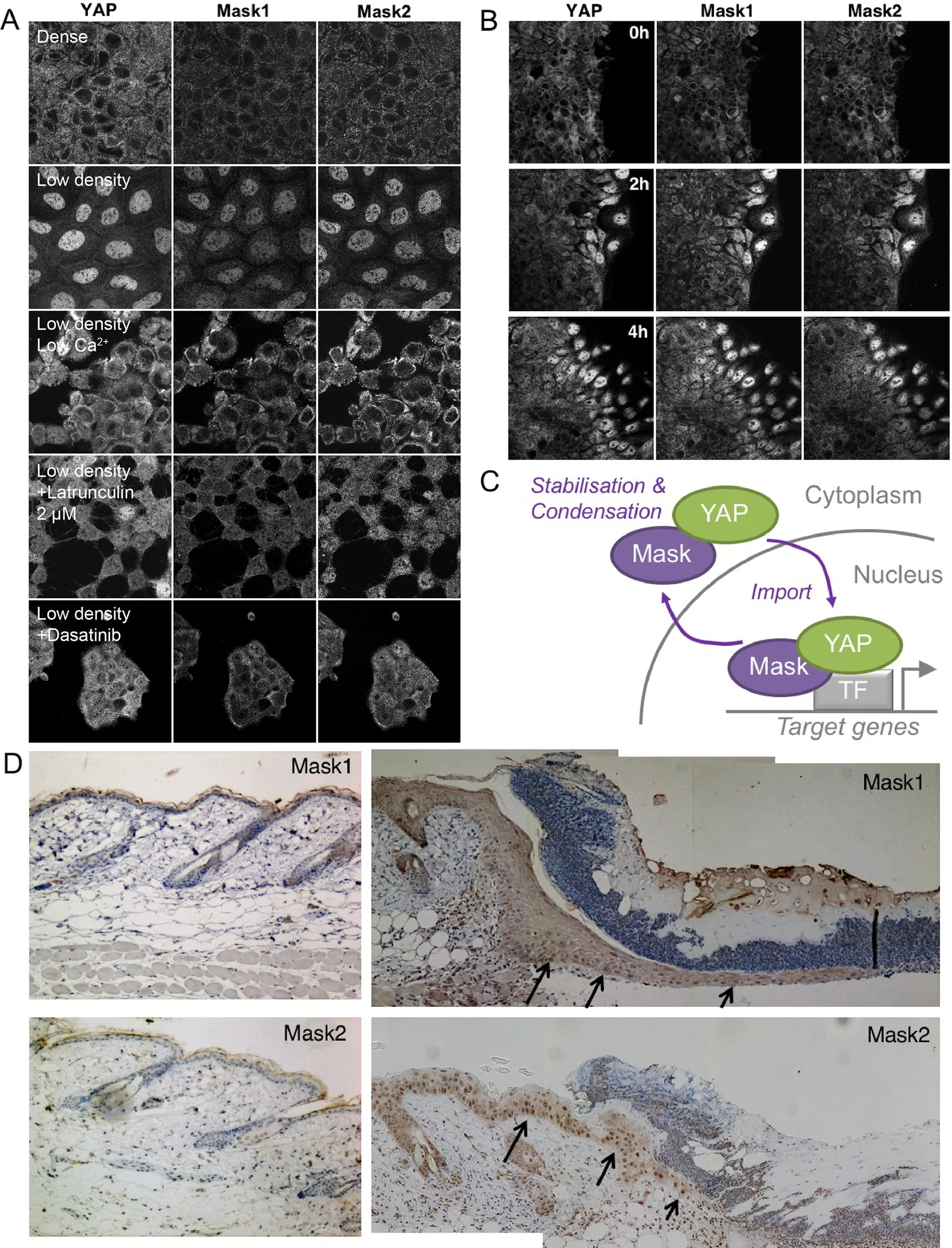


The originally published Figure 8 is shown for reference:

**Figure 2:**
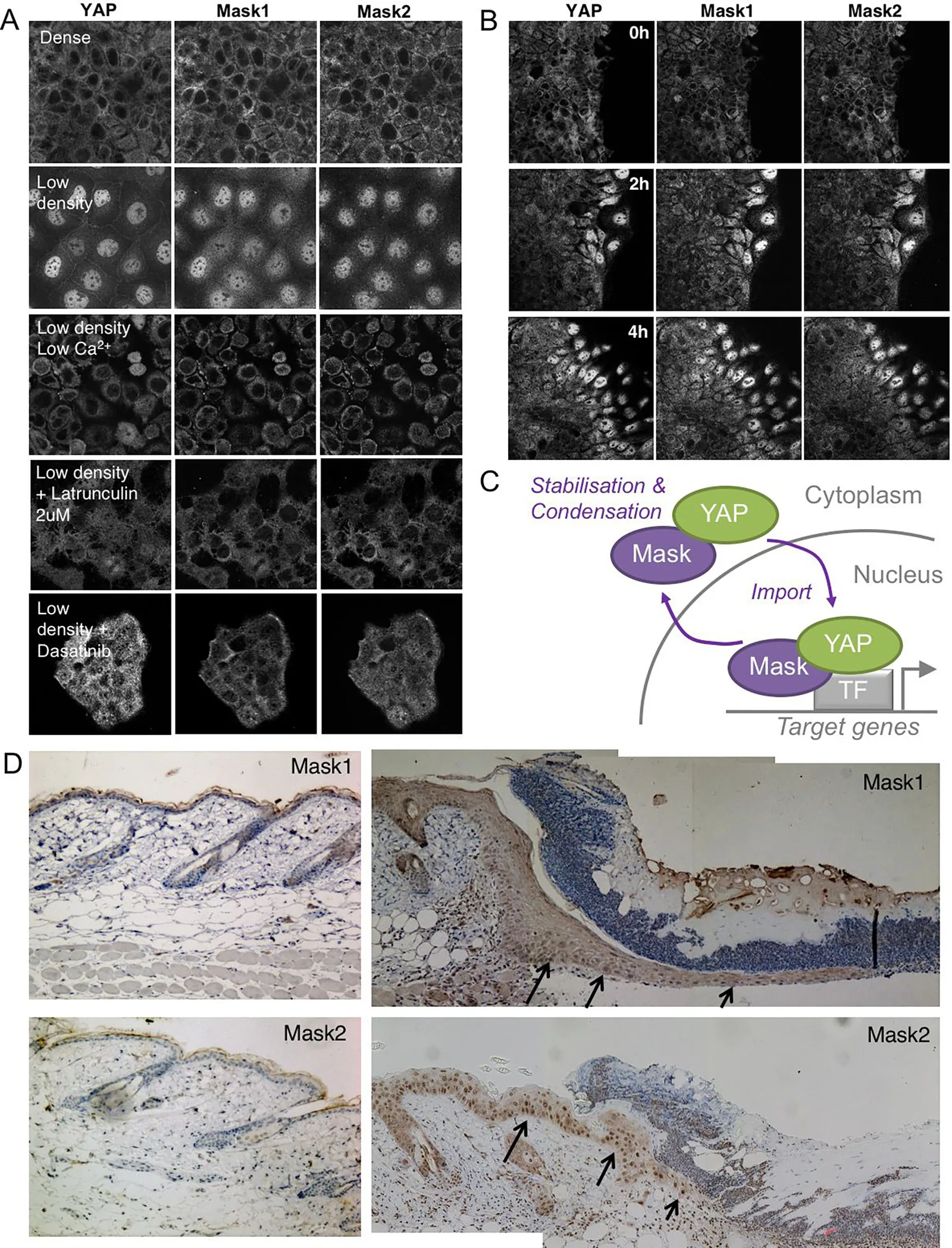


During the revision process, we also identified two minor oversights in Figure 7. First, the left panel of Figure 7A is an inset taken from the image shown in Figure 2B. This relationship should have been stated explicitly in the Figure 7A legend but was inadvertently omitted. Second, the label in Figure 7A contained a typographical error and should have read “stage 13” rather than “stage 14”. A reference to Figure 2B has now been included in Figure 7 legend and the typographical error has been corrected.

Corrected text (underlined) in Figure 7 legend:

**Figure 7. Endogenous Yki/YAP and Mask co-localise in intracellular granules in both nucleus and cytoplasm**. (A) Yki-GFP exhibits a granular appearance in both nucleus and cytoplasm of flattened *Drosophila* follicle cells (inset from Figure 2B). Yki-GFP also exhibits a granular appearance in the cytoplasm of S2 cells. (B) Co-localisation of endogenous Yki and Mask in nuclear and cytoplasmic granules of S2 cells. (C) Co-localisation of endogenous YAP and Mask1 in nuclear and cytoplasmic granules of HEK293 cells. (D) Schematic diagram of multivalent interactions between YAP binding partners and the possible formation of clusters (hubs/granules) in either the cytoplasm or nucleus.

Original text in Figure 7 legend:

**Figure 7. Endogenous Yki/YAP and Mask co-localise in intracellular granules in both nucleus and cytoplasm**. (A) Yki-GFP exhibits a granular appearance in both nucleus and cytoplasm of flattened *Drosophila* follicle cells. Yki-GFP also exhibits a granular appearance in the cytoplasm of S2 cells. (B) Co-localisation of endogenous Yki and Mask in nuclear and cytoplasmic granules of S2 cells. (C) Co-localisation of endogenous YAP and Mask1 in nuclear and cytoplasmic granules of HEK293 cells. (D) Schematic diagram of multivalent interactions between YAP binding partners and the possible formation of clusters (hubs/granules) in either the cytoplasm or nucleus.

The corrected Figure 7 (label in panel A updated) is shown here:

**Figure 3:**
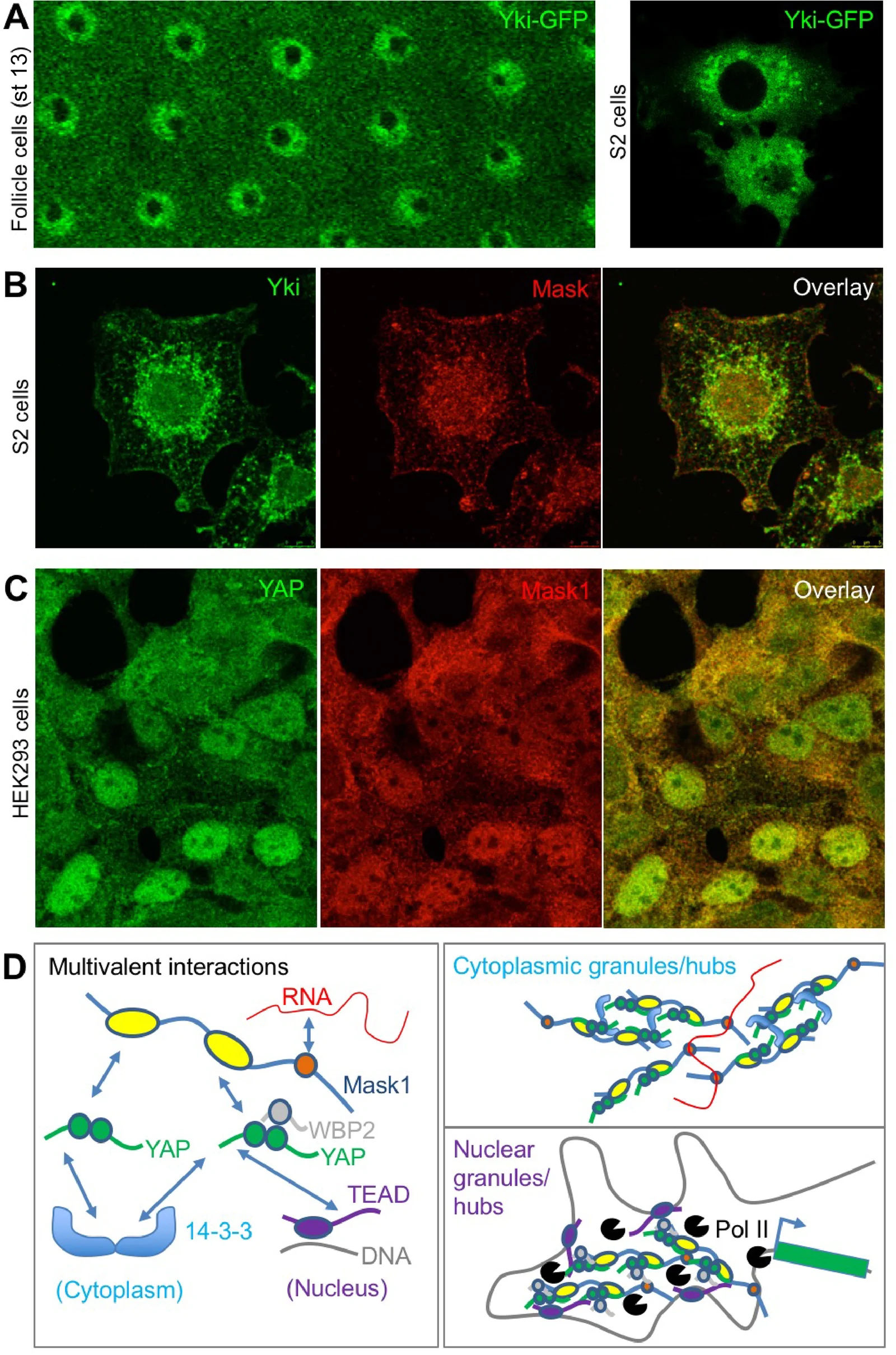


The originally published Figure 7 is shown for reference:

**Figure 4:**
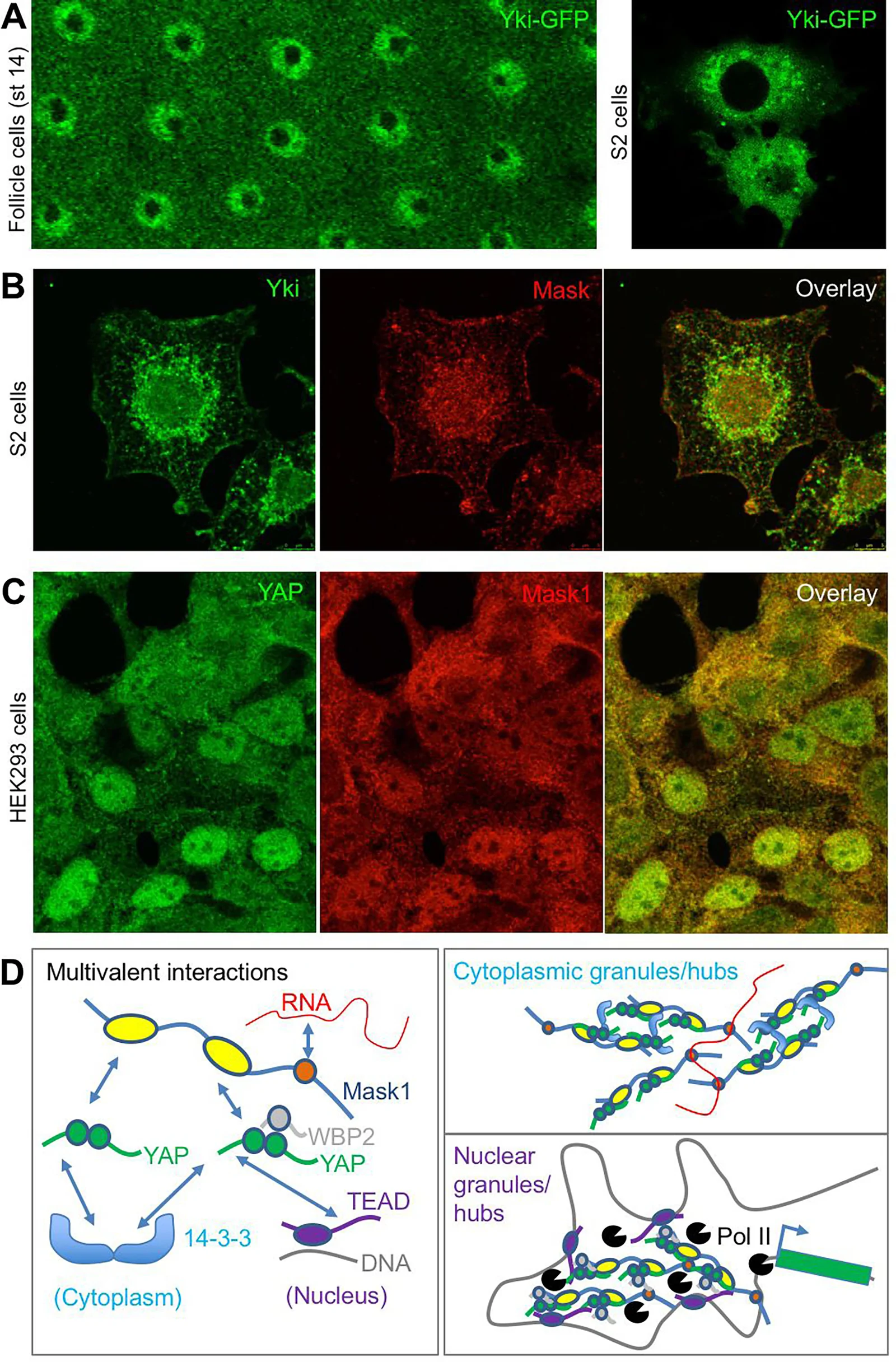


The article has been corrected accordingly. The corrections have been approved by all authors except BJT who could not be reached.

